# Health impacts from living near a major industrial park in Oman

**DOI:** 10.1186/s12889-015-1866-3

**Published:** 2015-06-02

**Authors:** Adil Al-Wahaibi, Ariana Zeka

**Affiliations:** Institute of Environment, Health and Societies, Brunel University London, Kingston Lane, Uxbridge, Middlesex United Kingdom UB8 3PH

**Keywords:** Environmental Exposure [N06.850.460.350], Primary Health Care [N04.590.233.727], Respiratory Tract Infections [C08.730], Hypersensitivity [C20.543]

## Abstract

**Background:**

Oman is heading towards heavy industrialisation with rapid establishment of new industrial parks. One of these, the Sohar Industrial Zone (SIZ) started to operate in 2006 and includes many industries that potentially affect local air quality and the health status of its surrounding residents. The study aim was to assess the health effects in a population of ≥20 years old, living in the residential area around the SIZ.

**Methods:**

Area-specific health care visits data for acute respiratory diseases (ARD), asthma, conjunctivitis and dermatitis were obtained for the period between January 1, 2006, and December 31, 2010. Exposure was defined as distance from the SIZ to determine high, intermediate, and control exposure zones (≤5, >5–10, and ≥20 km from the SIZ respectively). Generalized additive models were used to model age and gender adjusted monthly health events for the selected diseases, adjusted for age and gender-specific population smoking prevalence. The high and intermediate exposure zones were later combined in the models because of their similarity of effects. Exposure effect modification by age, gender and socio-economic status (SES) were examined.

**Results:**

Living within the high and intermediate exposure zones was associated with a greater risk ratio for ARD (RR: 2.02; 95 % CI: 1.88–2.17), asthma (RR: 3.61; 95 % CI: 2.96–4.41), conjunctivitis (RR: 2.83; 95 % CI: 2.47–3.24), and dermatitis (RR: 2.11; 95 % CI: 1.86–2.39), compared to the control exposure zone. Greater exposure effects were observed amongst ages ≥50 years and lower SES groups.

**Conclusion:**

This is the first study carried out in Oman to assess the link between environmental exposure and health. These findings hope to contribute to building up evidence for environmental health and sustainable development policy in the country.

**Electronic supplementary material:**

The online version of this article (doi:10.1186/s12889-015-1866-3) contains supplementary material, which is available to authorized users.

## Background

In the last few decades, the global trend for industrial development has migrated from developed to the less developed countries. As estimated by the World Bank, developing countries have more than doubled their energy production and consumption during the period from 1990–2011 compared to the developed countries [1]. This shift in energy production accompanied by the lack and inefficiency of environmental health policies, has also contributed to the geographic shift of the burden of environmentally related morbidities and mortalities [2]. A recent report of the World Health Organisation (WHO) estimates 25 % of mortalities in developing countries are environmentally related; this estimate is 17 % in developed countries [3].

Oman, through reforms in social and economic policies, which took place in the 1970s, started the development of different societal sectors including industries. The country has recently embraced a rapid industrialization trend to enrich its economy and decrease its dependency in fossil fuel [4]. As a result, many industrial parks including petrochemical industrial complexes were started in Oman, one of which is the Sohar Industrial Zone (SIZ). This industrial zone was established in 2006 and consists of two main industrial regions: Sohar Industrial Port (SIP) and Sohar Industrial Estate (SIE). Covering an area of 2058 hectares, SIP contains a wide range of petrochemical industries and an iron smelter. An oil refinery and a polypropylene plant started to operate in 2006, subsequently followed by two major industries: a power company and a methanol industry in 2007. A formaldehyde plant and a urea industry were established between 2008 and 2009; an iron smelter and an aromatics plant were appended in 2010. New expansions and developments will be established soon including more heavy industries and an airport [5]. SIE, which was built on 2100 hectares, is situated five kilometers southwest of SIP. Besides other complementary industries, Sohar Aluminium which started in 2008, is the most important industry in SIE for its size of investment [6]. Such industries potentially emit air pollutants and many environmental toxins such as sulphur dioxide (SO_2_), nitric oxides (NO_x_) and volatile organic compounds (VOC) [7].

Consistent epidemiological evidence elsewhere shows adverse health effects of air pollution, including increases in morbidity and mortality from respiratory and cardiovascular causes [8]. Several studies have also shown that there is a link between living near industrial complexes and occurrence of adverse health outcomes [9–11]. SIZ is situated near a heavily populated area, raising great concerns for community adverse health effects of its surrounding residents. Only two studies have assessed the environmental effect of SIZ in their surrounding area, suggesting adverse environmental conditions [12, 13]; one of these studies was conducted before the start of intensive industrial establishments in the area [13]. There has been no study of the health impacts of this industrial development, and more generally, in other similar developments in Oman. Hence, the aim of this study was to evaluate the acute health effects in an adult population living near the Sohar Industrial Zone.

## Methods

### Health and population data

As SIZ is situated between Liwa and Sohar provinces, health data on primary, secondary and tertiary care visits of the local population aged ≥20 years old were obtained from the Omani Ministry of Health (MoH). Data were gathered from the national Al-Shifa electronic health recording system from eight state health institutions in these provinces, for the period of January 1, 2006, and December 31, 2010. Along with Sohar city, the capital of Sohar province, this data represented a total of 59 villages. Data from private institutions were not available on this electronic system. The information obtained for each patient visit included: patient’s consultation date, unique identification number, date of birth, village, health institution, and diagnosis determined by the *International Classification of Diseases 10*^*Th*^*Revision* (ICD-10 code). Ethical approval for the study was obtained from the Omani MoH and Brunel University Ethics Committee.

Based on previous evidence on the effects of air pollution on morbidity outcomes and availability of data, we selected to study respiratory and allergic diseases.

Diseases of the respiratory system included: acute respiratory diseases (ARD) defined as upper (ICD-10: J0-J06), other acute lower respiratory infections (ICD-10: J20-J22), and pneumonia (ICD-10: J12-J18); and asthma (ICD-10: J45 and J46). Allergic diseases included disorders of the conjunctiva (ICD-10: H10-H13), and dermatitis - including eczema (ICD-10: L20-L30 and ICD-10: L50-L54, respectively). Due to the small number of visits for cardiovascular diseases, stroke and chronic obstructive pulmonary disease, data were insufficient for the analysis. The low number of these diseases, which mostly affect the ≥60 years old age group, potentially reflects the fact that the Omani population is young, with only 4.3 % of the population aged over 60 years [14].

Demographic data for area-specific population by age and gender, educational and occupational statuses were obtained from the Omani national census of 2010. The area-specific population was used as an offset for the models. Education and occupational status were used to construct the socio-economic status (SES) indicators for each village. Age and gender-specific smoking prevalence data were derived from a study done by Al Riyami et al. [15], and the Global Youth Tobacco Survey (GYTS-Oman) [16]. Meteorological data such as daily wind speed, wind prevailing direction and temperature were obtained from the Omani Department of Meteorology for the period of January 1, 2006, and December 31, 2010.

#### Definition of the events

To assess health effects of living in proximity to SIZ, two case definitions were used for each of the selected diseases: incidence (new) and follow-up cases. Previous epidemiological studies examining respiratory morbidity in adults define a new case and duration of ARD differently. While some studies defined the new event as a patient coming after one disease-free day [17], others defined this duration as 3 days [18], one week [19] and 1 month [20]. Several published studies considered a new asthmatic attack as an attack occurring after 1 year from the previous one [21]. However, Eisner et al. defined this period as one-month free attack in studying the effects of passive smoking on adult asthma [22]. Previous clinical and epidemiological definitions of a new case of allergic disease vary from 10 days [23] up to 1 year [24]. Using this previous epidemiological evidence, we defined a new case as any unique patient visit occurring 1 month after the first visit, including this first visit, for all disease definitions. The use of the one-month lag between visits to define new cases in our study also ensured sufficient number of events to improve the study efficiency, and decrease the possibility of counting follow-up visits as new events. Follow-up visits were defined as any patient’s visit occurring within the one-month period, between two new events.

### Exposure classification

Due to lack of air pollution monitoring or measurements in the area, we employed a proximity method to classify the exposure of the villages around SIZ. Villages were classified according to their distance from the source of pollution. The oil refinery was used as a landmark source from which to estimate the distance, determined by this industry being the main source of exposure in SIZ and its early operation date. The proximity approach is widely used in many influential environmental epidemiology studies [10, 11, 25–29]. In addition, this method is used frequently in many environmental investigation studies such as in the environmental impact assessment and environmental justice studies [30]. For example, the United States Environmental Protection Agency (US EPA) has used this approach in its environmental risk assessment framework [31].

To determine the most appropriate definition of the minimum distance for exposure, we used previously published evidence. In several epidemiologic studies, the designation of the minimum proximity distance for exposure classification was mostly arbitrary [26, 32, 33]. However, one study defined the distance depending on residents’ odor complaints [27], while others defined it by using environmental sampling [34, 35]. The threshold distance used in the studies assessing the effects of petrochemical industrial complex ranged from 3 km [10] to 20 km [11], whereas two studies used a 20 km distance for the metal smelters [27, 32]. We also reviewed several examples of international housing policies, which determined the minimal safe distance for residences from such industrial areas as >2 km (Additional file 1).

**Fig. 1:**
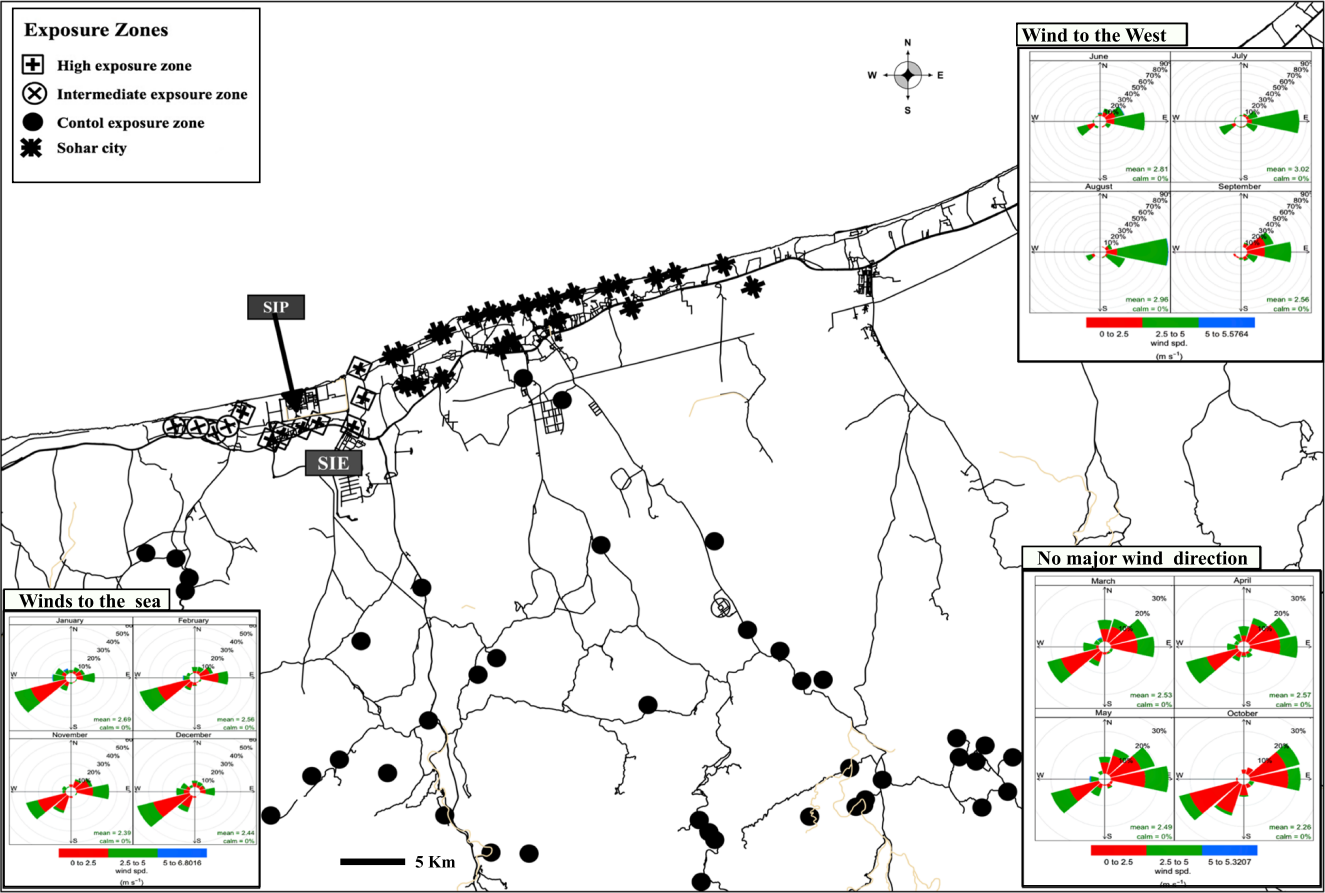
Exposure classification of the study area*. * The prevailing wind direction is illustrated by wind roses with the corresponding direction. The approximate location of the villages is illustrated. SIP: Sohar Industrial port; SIE: Sohar Industrial Estate. Villages are only located north to SIE

Based on the above discussion and to increase the power of the study, an incremental distance of 5 km from the refinery was chosen to determine exposure zones. We defined four exposure zones: high, for those living within ≤5 km; intermediate, living within >5–10 km; and control exposure zone, as living ≥20 km from the refinery. No villages were located between 10 and 20 km from the refinery; hence, this distance was not represented in the analyses. This study only included data from state health institutions, likely contributing to relatively smaller number of cases from Sohar city. The latest statistics from MoH revealed that out of the fifty-one private health clinics found in the studied area, 48 (94 %) of these clinics are located in Sohar city, including 20 medium size health complexes and two private hospitals [36]. In contrast, the three remaining private clinics, which were located in other zones, comprised of only one medium sized and two small private clinics. Hence, to ensure minimal contribution of selection bias, Sohar city was excluded from the analyses (Fig. 1).

To validate the proximity approach-based exposure classification, we constructed monthly wind roses of the area for the entire study period. The wind pointed to the west in the summer and in September, to the northeast, and towards the seaside in November and in winter, and had no major direction in spring and in October (Fig. 1). This indicated a possible similarity in the pollutant patterns between the high and intermediate exposure zones during the summer and the month of September.

Also, to verify our exposure classification distances, we carried out dispersion modeling using limited emission data for SO_2_. SO_2_ emission data were obtainable for the oil refinery for only the first 15 days of June 2008. To run the dispersion model, two main sets of data were needed: the SO_2_ emission data, obtained from AbdulWahab et al. [37], and meteorological data produced using the high-resolution Meteorological Mesoscale Model (MM5) and terrain data [38]. Dispersion model predicted SO_2_ concentration showed a relative two to four times change for distances from 5 to 10 km, supporting our 5 km incremental distances used to define exposure zones.

### Statistical analysis

Monthly health care visits were modeled using a generalized additive model (GAM) in R software. Modeling diagnostics, including Akaike’s Information Criterion, over-dispersion, and influence graphs suggested the use of a negative binomial distribution to count for data over-dispersion. Time, as month number, was defined as a continuous variable and smoothing functions were applied to capture underlying non-linear seasonal and longer-term trends of the selected diseases. Positive autocorrelation was detected in the data and was controlled by an autocorrelation structure of order 1 (corAR1). Exposure zones (with control zone as a reference), age category (≥20–49, ≥50 years old) and gender were all coded as factors in the model. The logarithm of gender and age group-specific population data were added as an offset in the model.

Smoking was not equally distributed in the study population. Latest statistics in Oman show that the average smoking prevalence amongst males was 13 %; this was only 0.8 % amongst the females [15, 16]. Because of the large difference of smoking prevalence between gender groups in our study population, smoking was controlled for in the analyses. Other potential confounders including daily temperature, season, and individual year, were statistically tested and found not to change the exposure-response estimate by more than 10 %, thus not considered further. The use of time smoothing function in the model potentially controlled for other time-varying confounders.

Risk ratios (RR) for the different exposure zones were calculated as [exp^(*β1*)^] and the 95 % confidence interval (CI) levels were calculated using the formula: exp ^(*β1*±1.96 X SE)^.

All statistical analyses were conducted using R software version 3.1.2. Modeling was carried out using the *GAM* function (*mgcv and MASS*).

Because of the similarities of the RR between the high and intermediate exposure zones, we carried out further analysis combining these two exposure zones. The feasibility of this combination was also suggested by the similar distributions of pollutant dispersion observed by wind roses and dispersion model.

#### Sensitivity analysis

To ensure that our models were capturing exposure effects, we carried out additional analyses for follow-up visits of ARD, asthma, all musculoskeletal diseases (MS), and gastrointestinal diseases (GID). The latter two diseases are not evidenced to be affected by air pollution. Monthly follow-up visits of MS and GID were modeled using the same model structure as for other disease definitions, but with the total exposure zone population as an offset due to lack of age and gender-specific information for these groups.

We assessed for effect modification by gender, and categories of age and indices of SES. Models were stratified by gender, and age in two categories: ≥20–49 and ≥50 years old. Indices of SES were available for all the villages in the study. We used the percentages of the population with ‘no education’, ‘high education’ and ‘employment’ in each village to determine SES. The ‘high education’ percentage was defined as the proportion of individuals in each village that had received a bachelor degree and above. Two levels of SES were then defined by stratifying the ‘no education’, ‘high education’ and ‘employment’ proportion distributions across all villages into two equal strata: ≤, and >50 %.

## Results

During the study period, the total number of visits for the selected diseases was 74,047. The total population-at-risk was 27,688 (Table 1). Table 1 also shows the greater number of males in the population of age groups ≥20–49 years old. This was more evident in the high exposure zone. However, for each exposure zone, the proportion of the event counts for males and females were comparable. A noticeable small percentage of event counts occurred in the ≥50 year old group in all exposure zones, except for asthma.

**Table 1:** Descriptive characteristics of the studied population including the total population-at-risk in the exposure zones and the total number of monthly events, classified by age group and gender

Study population	Age Category	Gender	Exposure Zone		
			High	Intermediate	Control
Total population at risk (%)^a^	≥20–49 years	F	3239 (26.7)	3159 (35.2)	2076 (31.5)
		M	7607 (62.7)	4498 (50.2)	3233 (49.0)
	≥50 years	F	576 (4.7)	598 (6.7)	616 (9.3)
		M	705 (5.8)	712 (7.9)	669 (10.1)
Socio-economic indicators^b^	Mean ‘no education’% (SD)		66.8 (21.3)	52.9 (4.5)	47.6 (9.7)
	Mean ‘high education^c^ ’% (SD)		1.7 (2.3)	3.8 (2.4)	10.4 (13.7)
	Mean ‘employment’% (SD)		83.4 (10.2)	76.4 (3.1)	67.7 (11.3)
ARD^d^ monthly visits (%)	≥20–49 years	F	11,814 (42.9)	8892 (40.5)	3595 (39.3)
		M	9980 (36.3)	9116 (41.5)	3354 (36.7)
	≥50 years	F	2830 (10.3)	1802 (8.2)	1160 (12.7)
		M	2889 (10.5)	2146 (9.8)	1035 (11.3)
Conjunctivitis monthly visits (%)	≥20–49 years	F	869 (30.5)	840 (32.7)	275 (31.8)
		M	981 (34.5)	974 (37.9)	256 (29.6)
	≥50 years	F	415 (14.6)	314 (12.2)	155 (17.9)
		M	581 (20.4)	439 (17.1)	179 (20.7)
Dermatitis monthly visits (%)	≥20–49 years	F	1105 (39.0)	665 (31.0)	404 (41.5)
		M	963 (34.0)	965 (45.0)	260 (26.7)
	≥50 years	F	333 (11.8)	196 (9.1)	118 (12.1)
		M	433 (15.3)	320 (14.9)	191 (19.6)
Asthma monthly visits (%)	≥20–49 years	F	461 (33.9)	502 (35.2)	106 (25.4)
		M	246 (18.1)	271 (19.0)	122 (29.3)
	≥50 years	F	223 (16.4)	216 (15.1)	98 (23.5)
		M	430 (31.6)	437 (30.6)	91 (21.8)

^a^Percentage to the total counts in the exposure zone. ^b^Showing the mean percentage of SES category for the villages of each exposure zone with the standard deviation. ^c^High education: individuals with bachelor degree and above. ^d^Acute respiratory Diseases

**Fig. 2:**
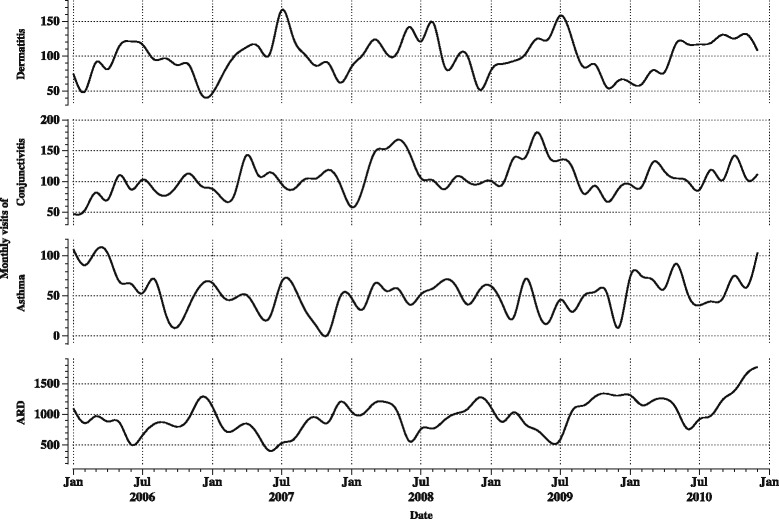
Monthly event counts of acute respiratory diseases, asthma, conjunctivitis and dermatitis*. *Monthly events for each selected cause were summed for all exposure zones and plotted against the date of visit

Figure 2 shows the monthly event counts of ARD, asthma, conjunctivitis, and dermatitis with clear seasonal variability. For ARD, more monthly event counts were noted in autumn and winter, whereas for dermatitis and conjunctivitis these events were higher in spring and summer.

In Table 2, associations for all disease events and exposure definitions are presented. The results show around two folds greater effects for all diseases in the high and intermediate exposure zones when compared to the control exposure zone. This similarity in the trend suggested the feasibility of combining the two zones into one exposure zone of ≤10 km radius from the refinery.

**Table 2:** Multivariate Analysis^a^ of acute respiratory diseases, asthma, conjunctivitis and dermatitis incidence^b^. Comparison between the high and intermediate exposure groups

Studied disease	High Exposure Zone RR (95 % CI)	Intermediate Exposure Zone RR (95 % CI)
ARD	2.30 (2.11–2.52)	1.83 (1.67–2.00)
Asthma	3.46 (2.80–4.29)	3.76 (3.04–4.66)
Conjunctivitis	2.92 (2.52–3.38)	2.50 (2.15–2.89)
Dermatitis	2.27 (1.97–2.62)	1.75 (1.52–2.03)

^a^Adjusted for time trend and smoking prevalence

^b^Age and gender standardized according to census population figures for 2010

*RR*, risk ratio; *CI*, confidence interval; *ARD*, acute respiratory diseases

Table 3 shows the RR in the combined exposure zone when compared to the control zone, overall and stratified by gender and age groups. Positive associations were found for all selected diseases in the combined exposure zone. Stratifying the data by age category showed around 30 % change in the effects amongst ages ≥50 years, compared to the ≥20–49 years old age group for ARD and asthma. We found no differences in the effects by gender.

**Table 3:** Multivariate Analysis^a^ of acute respiratory diseases, asthma, conjunctivitis and dermatitis incidence^b^. Results are also stratified by age category and gender

Stratification	Combined^c^ RR (95 % CI)
ARD	
Overall	2.01 (1.87–2.17)
Males	1.86 (1.70–2.05)
Females	2.02 (1.84–2.21)
≥20–49 years	1.75 (1.61–1.90)
≥50 years	2.27 (2.07–2.49)
Asthma	
Overall	3.60 (2.95–4.40)
Males	2.47 (2.01–3.04)
Females	2.85 (2.34–3.46)
≥20–49 years	1.93 (1.58–2.35)
≥50 years	3.73 (3.01–4.63)
Conjunctivitis	
Overall	2.82 (2.46–3.23)
Males	2.49 (2.17–2.87)
Females	2.37 (2.05–2.73)
≥20–49 years	2.22 (1.95–2.52)
≥50 years	2.73 (2.34–3.18)
Dermatitis	
Overall	2.09 (1.84–2.38)
Males	2.06 (1.78–2.39)
Females	1.79 (1.55–2.08)
≥20–49 years	1.78 (1.55–2.04)
≥50 years	2.18 (1.85–2.56)

^a^Adjusted for time trend and smoking prevalence

^b^Age and gender standardized according to census population figures for 2010

^c^Including high and intermediate exposure zones, control exposure zone as reference

RR risk ratio; CI confidence interval; ARD acute respiratory diseases

Table 4 shows the results of exposure-ARD visits associations by categories of SES indices. A 30 % greater effect was observed for villages with lower proportions of ‘high education’ and ‘employment’.

**Table 4:** Multivariate Analysis^a^ of acute respiratory disease^b^. Classification by three SES parameters

SES classification	RR^c^ (95 % CI)
≤50 % ‘no education’ strata	1.90 (1.80–2.01)
>50 % ‘no education’ strata	2.11 (1.95–2.28)
≤50 % ‘high education’ strata	2.21 (2.06–2.38)
>50 % ‘high education’ strata	1.73 (1.63–1.84)
≤50 % ‘employment’ strata	2.33 (2.20–2.48)
>50 % ‘employment’ strata	1.62 (1.51–1.73)

^a^Adjusted for time trend and smoking prevalence

^b^Age and gender standardized according to census population figures for 2010

^c^Risk ratio of combined exposure zones in reference to control exposure zone

RR risk ratio; CI confidence interval

Results of the model analyses for associations of the monthly follow-ups for ARD, asthma, MS and GID are given in Table 5. Excess risk was observed for follow-up frequencies in the combined exposure zones when compared to the control zone for ARD and asthma. These effects were comparable to the associations for ‘new events’. We did not observe any association between exposure and MS and GID follow-up counts.

**Table 5:** Multivariate Analysis^a^ of acute respiratory diseases, asthma, MS and GID follow-up^b^ frequencies^c^

	Combined^d^ RR (95 % CI)
ARD	1.74 (1.52–1.98)
Asthma	2.23 (1.92–2.60)
MS	0.94 (0.86–1.02)
GID	0.94 (0.84–1.04)

^a^Adjusted for time trend and smoking prevalence

^b^Follow-ups were defined as any patient’s visit to the doctor occurring within the defined period for the ‘new event’ definition

^c^Crude exposure zone population was used as the model offset according to census population figures for 2010

^d^Including high and intermediate exposure zones, control exposure zone as reference

*RR*, risk ratio; *CI*, confidence interval; *ARD*, acute respiratory diseases; *MS*, All musculoskeletal diseases; *GID*, Gastrointestinal diseases

## Discussion

This study was carried out to examine the acute health impacts in adults living near a major industrial park in Oman. After adjusting for age, gender, time trend and smoking prevalence, findings suggested that living closer to the industrial park increased the risk of health care visits from acute respiratory tract diseases, asthma, conjunctivitis and dermatitis two to three folds when compared to the control zone. The use of non-parametric time-series models allowed for the adjustment of temporal changes such as seasonal, long, and short-term variations of events in the classified area.

In our study, we did not have any air quality data for the study area or any information on the actual emissions of the industries that would enable us to construct full dispersion models. This led us to use the proximity to source method to classify exposure for the population living around SIZ. The proximity approach has been utilized frequently in many influential epidemiological and public health studies, particularly in the assessment of health effects around petrochemical industrial complexes [10, 11] and heavy metal smelters [32, 39]. In addition, the approach has been used by governmental environmental protection bodies in their policies, such as the US EPA [31] and the United Kingdom’s Health and Safety Executive [40]. Because this method is easy, quick and economic, it is used frequently to assess health effects from industrial chemical spillages [41] and environmental justice studies [42].

The combination of the two exposure zones into one zone of ≤10 km suggested the widespread health effects of the industrial park. This extent of health effects is possibly due to the unfavorable geography of the area, also noted in a recent meteorological study by Al-Khadouri et al. [43]. The authors showed that the air quality in SIZ is prone to stagnation and recirculation, with no single episode of ventilation. These characteristics facilitate a stagnant pollutant mass in the area that was shown, by numerical simulation, to reach >10 km inland.

Supporting our study results, epidemiological studies have shown that living near a petrochemical complex is associated with around two folds increase in acute irritant symptoms of the respiratory tract and eye, and asthma [44–46]. A study in China showed that persistent toxic substances from these industries have doubled the risk of developing dermatitis in people living in proximity [47]. According to a Brazilian study, living near aluminium smelters has been associated with four folds increase respiratory disease admissions [48], whereas a Canadian study showed that living near iron smelters has been associated with acutely declining lung functions [49].

Petrochemical industrial complexes, like in SIZ, are sources of SO_2_, NO_x_ and VOC [7]. These gases are shown to cause acute irritant effects of the lining of the respiratory tracts eyes and the skin [50]. The aluminum smelter potentially emits many hazardous chemicals to the atmosphere such as aluminum compounds, SO_2_ and fluoride compounds; iron smelters emit SO_2_, NO_x_ and heavy metals [7].

In our study, living within proximity of exposure source showed a greater risk of ARD and asthma amongst the ≥50 years old age group, and this finding may suggest increased vulnerability amongst the older age group [51]. The gender stratified exposure-disease associations, specifically for asthma and conjunctivitis, did not correspond to the overall RR. One explanation is the unequal distribution of smoking prevalence amongst these two groups in Oman [15, 16]. The effect of the unequal smoking prevalence between the gender groups was also verified after performing another modeling excluding the smoking prevalence which showed corresponding gender stratified exposure-disease associations with the overall RR (Additional file 2).

Some suggestion of unequal effects by SES group was observed, with disadvantaged showing greater effects of living in proximity to exposure source for events of ARD and asthma. This is potentially due to other unfavorable circumstances associated with disadvantaged SES such as general health habits (smoking and alcohol drinking), occupational and other environmental factors including disadvantaged air quality due to living near industrial sources [52].

To ensure that we were adequately capturing exposure effects, we compared the follow-up frequencies of the ARD, asthma, MS and GID. Unlike the effects in the ARD and asthma, which showed increased risks associated with exposure, the associations for MS and GID suggested no differences in the follow-up frequency between exposure zones. Hence, this confirmed that our exposure assessment approach could differentiate exposure effects.

The results showed that the number of young males (≥20–49 year old) was greater compared to the young females. This disproportionate gender distribution in the area was also observed for the entire Omani population pyramid, using census 2010. This phenomenon could be explained by the high number of working male expatriates in this age group as evident by the Omani population pyramid, also supported by higher relative proportions of young males in exposure zones compared to control zone.

Few limitations of this study need discussion. First, the use of a proximity method for exposure classification is subject to misclassification bias. This bias arises when there is uncertainty in the outline of the limit of exposure zones, as pollutants do not respect boundaries [53]. Therefore, villages within each exposure zone may experience heterogeneity in exposure levels. However, in our exposure classification, we used previous evidence from epidemiological and policy studies [10, 11] which supported the incremental distance used here. Additionally, analyses of wind roses and dispersion model confirmed the validity of the selected distances and have contributed to minimize this bias. Second, morbidity data likely have a less complete representation of the disease in a specific area, unlike the self-defining mortality data. This is because morbidity data rely on patient’s threshold to seek medical help from the primary health care centers; this threshold is determined by the patient, his family, and the surrounding society’s perception and attitude to the disease, and accessibility to health institutions [54]. While more perceptive factors could not be factored in this study, the uniform distribution of the health institutions in the area ensured an easy access to health care providers for the population under study. Third, only the state-related and not the private health institutions were considered in this study, which might introduce a selection bias. However, the low number of private clinics in the exposure zones, after excluding Sohar city from the analyses, minimized the occurrence of this bias. Last, morbidity data can be affected by the method used to distinguish between the new cases from the follow-ups. The definitions used in our study were determined based on previously tested comprehensive clinical and epidemiological definitions.

Our study is the first environmental epidemiologic study carried out in Oman, which investigates the health impacts of the country’s rapid industrial development. Our findings suggest that the inevitable economic growth and rapid industrial development of Oman could, potentially, have adverse population health effects. This would signify the need for a more sustainable development in the country by implementing a powerful environmental health system to balance this rapid development. The necessity for an environmentally and socially sustainable development is being recognised in many rapidly developing economies around the world. For example, China has suffered tremendously from air pollution with an estimated 2.3 billion dollars annual loss due to morbidities and mortalities related to air pollution [55]. This triggered the Chinese government to implement more policies regarding environmental health. In 2004, the WHO has estimated that the burden of environmental diseases in Oman was about 17 % of the total burden of diseases [56]; this estimate will likely increase in relation to prospective planned industrial developments and urbanization trends in the country. Hence, it is essential that future policies should consider improvements of public and environmental health system and tracking, along with industrial development.

## Conclusion

This study suggested an increase in adverse acute health effects in an adult population living near the newly developed Sohar industrial zone. As this is the first environmental epidemiologic study carried out in Oman, we hope that these findings will contribute to increasing awareness and further research on environmental health issues. These findings will hopefully encourage the initiation of preventive actions and public health intervention programs in the country.

### Availability of supporting data

Health data are available from the Omani Ministry of Health only after Institutional Data Access/Ethics Committee for researchers who meet the criteria for access to confidential data.

## Additional files

Additional file 1:
**Examples of international housing policies for the minimum acceptable distance from industry to residential areas.**
Additional file 2:
**A table of Multivariate Analysis of asthma and conjunctivitis incidence adjusted for time trend but not for smoking prevalence.**

## Abbreviations

ARD: Acute respiratory disease
CALPUFF: California Puff
CI: Confidence interval
GAM: Generalized additive model
GID: Gastrointestinal diseases
ICD-10 code: International Classification of Diseases 10^th^ Revision
MoH: Ministry of Health
MM5: Meteorologic Mesoscale Model
MS: All musculoskeletal diseases
NB: negative binomial distribution
NO_x_: nitric oxides
RR: Risk ratios
SES: Socio-economic status
SIE: Sohar Industrial Estate
SIP: Sohar Industrial Port
SO_2_: Sulphur dioxide
SIZ: Sohar Industrial Zone
US EPA: United States Environmental Protection Agency
WHO: World Health Organisation.
