# Supplementary material for: Health impacts from living near a major industrial park in Oman
Source: BMC Public Health. 2015 Jun 2;15:524. doi: 10.1186/s12889-015-1866-3 (PMC4450448; doi:10.1186/s12889-015-1866-3)
Supplement: Additional file 1: — Examples of international housing policies for the minimum acceptable distance from industry to residential areas. [file 12889_2015_1866_MOESM1_ESM.pdf]

Additional file1. Example of international housing policies for the minimum acceptable distance from industry to residential areas.

Industry type	MAD	Government body	Country	Reference
Petrochemical industries	>2 km	EPA Victoria	Australia	[1]
Metal production	>1-2 km			
Aluminum production	>1.5-2 km	EPA Western Australia	Australia	[2]
Petroleum refining	>2 km			
Petroleum refining	>2 km	EPA South Australia	Australia	[3]
Aluminum by electrolysis	>2 km			
Petroleum refining	>2 km	Auckland council	New Zealand	[4]
Metal processing	>1km	Department of Environment	Malaysia	[5]

MAD: minimum acceptable distance
EPA: Environmental Protection Authority

1. EPA Victoria: *Recommended Separation Distances for Industrial Residual Air Emissions*. March. Victoria; 2013:1–17.

2. Segui C: *Guidance for the Assessment of Environmental Factors*. Western Australia; 2005:1–59.

3. Torr P, Watson D: *Guidelines for Separation Distances*. South Australia; 2005(consultation draft):1–23.

4. Wickham L: *Separation Distances for Industry*. 2012:1–44.

5. *Guidelines for the Siting and Zoning of Industry and Residential Areas*. 2nd edition. Malaysia; 2013:1–134.
