# Supplementary material for: Health impacts from living near a major industrial park in Oman
Source: BMC Public Health. 2015 Jun 2;15:524. doi: 10.1186/s12889-015-1866-3 (PMC4450448; doi:10.1186/s12889-015-1866-3)
Supplement: Additional file 2: — A table of Multivariate Analysis of asthma and conjunctivitis incidence adjusted for time trend but not for smoking prevalence. [file 12889_2015_1866_MOESM2_ESM.pdf]

Additional Table 1. Multivariate Analysis[a] of Asthma and conjunctivitis incidence[b]. Results are stratified by age category and gender.

Stratification	Combined[c] RR (95% CI)
**Asthma**	
Overall	3.87 (3.14-4.77)
Males	4.86 (3.61-6.55)
Females	3.00 (2.35-3.83)
≥20-49 years	2.25 (1.75-2.90)
≥50 years	3.86 (3.03-4.66)
**Conjunctivitis**	
Overall	2.90 (2.51-3.35)
Males	3.06 (2.45-3.84)
Females	2.65 (2.22-3.18)
≥20-49 years	2.26 (1.96-2.61)
≥50 years	2.93 (2.34-3.18)

[a] Adjusted for time trend.
[b] Age and gender standardized according to census population figures for 2010.
[c] Including high and intermediate exposure zones, control exposure zone as reference.
RR risk ratio; CI confidence interval.
